# Time to achieve steady state for an accurate assessment of resting energy expenditure in adolescents with healthy weight and obesity: A cross-sectional study

**DOI:** 10.20945/2359-3997000000450

**Published:** 2022-03-23

**Authors:** Isabela F. Soares, Fabrício Vasconcellos, Felipe A. Cunha

**Affiliations:** 1 Universidade do Estado do Rio de Janeiro Programa de Pós-graduação em Ciências do Exercício e Esportes Rio de Janeiro RJ Brasil Programa de Pós-graduação em Ciências do Exercício e Esportes, Universidade do Estado do Rio de Janeiro, Rio de Janeiro, RJ, Brasil; 2 Universidade do Estado do Rio de Janeiro Laboratório de Atividade Física e Promoção da Saúde Rio de Janeiro RJ Brasil Laboratório de Atividade Física e Promoção da Saúde, Universidade do Estado do Rio de Janeiro, Rio de Janeiro, RJ, Brasil

**Keywords:** Resting metabolic rate, kilocalories, oxygen uptake, reliability, CPX Ultima CardiO2, indirect calorimetry

## Abstract

**Objective:**

The present study investigated the time needed to achieve a steady state for an accurate assessment of resting energy expenditure (REE) in adolescents with healthy weight and obesity.

**Materials and methods:**

Thirty adolescents aged 12-17 years were assigned to a group with healthy weight (GHW; n = 12, body mass index [BMI] 22.5 ± 3.6 kg/m^2^) and another group with obesity (GO; n = 18, BMI 34.1 ± 5.2 kg/m^2^). Participants underwent test-retest reliability of REE assessment as follows: a) 24 h of abstention from physical exercise, soft drinks, or caffeine; b) fasting for ~12 h; c) acclimation period of 10 min; d) 30-min assessment in a supine position.

**Results and discussion:**

A significant change occurred during the 30 min in REE. Significant differences existed between consecutive means until the 20^th^ and 25^th^ min for the GHW and GO, respectively. Although significant differences between trials 1 and 2 were detected during the first 5-10 min of assessment, the REE for each 5-min time point exhibited high test-retest reliability across trials in both groups (intraclass correlation coefficients range 0.79-0.99).

**Conclusion:**

The following recommendations are provided to promote accurate assessment of REE among adolescents: a) initiate the REE assessment with 10 min of acclimation to decrease restlessness; b) determine REE for a minimum of 20 min if healthy weight and 25 min if obesity; c) determine REE for a further 5 min, with the average of this last 5 min of REE data being regarded as the REE.

## INTRODUCTION

Childhood obesity is a serious public health concern in developed and developing countries ( 1 ), and its overall prevalence has markedly risen in more than 70 countries by 20% from 1980 to 2015, which represents 107.7 million children worldwide ( 2 ). According to the Centers for Disease Control and Prevention, childhood obesity is defined as a body mass index (BMI) at or above the 95^th^ percentile for age and sex ( 3 ), and its long-term effects are directly associated with several comorbidities ( 4 ), a greater risk of adult obesity compared with counterparts with healthy weight ( 5 ), and a predictor of mortality in early adulthood ( 4 , 6 ).

Although obesity is a result of a complex combination of biological, developmental, environmental, behavioral, and genetic factors ( 1 ), the most common cause of weight gain is an imbalance between total energy intake and total daily energy expenditure, such that intake chronically exceeds energy requirements including basal metabolic rate (BMR), diet-induced thermogenesis, and activity-related energy expenditure ( 7 ). The BMR is the lowest resting energy expenditure (REE) required to maintain the body’s integrated systems and homeostasis; therefore, knowledge of REE is important in clinical applications because it accounts for the largest proportion of total daily energy expenditure ( *i.e.* , ~50-70%) ( 8 ).

Indirect calorimetry for the assessment of oxygen uptake (VO_2_) and carbon dioxide output (VCO_2_) has been widely used and accepted as the gold-standard method to determine the REE under strict conditions ( 9 ). In 2006, the Academy of Nutrition and Dietetics Evidence Analysis Library (AEL) published an early systematic review ( 10 ) to guide practitioners on the best procedures to promote accurate and reliable REE assessment in adults, which are summarized as follows: a) at least 6 h of fasting before the assessment to avoid the thermogenic effect of food; b) abstinence from coffee, alcohol, nicotine, or any other stimulant for at least 2 h before the assessment; c) a minimum rest period of 10 to 20 min before the assessment; d) restriction of physical activities for a minimum of 2 h for moderate activities and 14 h for strenuous activities; e) measurements made in supine or slightly elevated body posture; and f) minimum measurement duration of 10 min under steady-state (SSt) conditions, discarding the first 5 min of REE data. The SSt is defined as the first 5 min in which VO_2_ and VCO_2_ data achieve a coefficient of variation (CV) of 10% or less.

Although the systematic review by AEL ( 10 ) has progressed our understanding of the main criteria for the REE assessment in adults, it did not include the time needed to achieve SSt in VO_2_ and VCO_2_ data. An important question remains as to whether there is sufficient evidence that a short-term assessment of 10 min is a valid time to verify the attainment of SSt required for the determination of REE, especially among adolescents, which were not included in the systematic review of AEL ( 10 ). Some studies, for example, suggested that a short-term protocol of 10 min would be enough to provide a SSt condition and avoid individual restlessness in adults ( 11 ) and children ( 12 ). Others reported that following an acclimation period of 10 min, at least 30 min of REE assessment were needed to achieve the start of a SSt condition in adults ( 13 ).

Among the adolescent population, this particular aspect of REE determination remains totally unknown. Considering that puberty is a hormonal event marked by rapid growth and maturation into a short-term period that elicits changes in energy requirements and potentially increases the risks of energy imbalance ( 14 ), it is feasible to think that additional investigation is warranted to address the following research question: How long should the duration of the REE assessment be to achieve a SSt in adolescents with healthy weight and obesity? Thus, the aim of the present study was to identify the time needed to achieve a SSt for an accurate assessment of REE in adolescents with healthy weight and obesity. We hypothesized that after completion of an acclimatization period of 10 min at least 20 min are needed to obtain SSt conditions and to provide adequate test-retest reliability in adolescents, regardless of obesity status.

## MATERIALS AND METHODS

### Participants

Thirty adolescents (23 boys) aged 12 to 17 years volunteered for the study, recruited from schools in the city of Rio de Janeiro, RJ, Brazil. The participants were classified as obese when their BMI was above the 95^th^ percentile for age and sex ( 3 ). In order to participate in the study, the participants had to present sexual maturation above stage 3 for breast development, pubic hair, or genital size according to Tanner classification charts, or menarche (Tanner & Whitehouse, 1976). Girls and their mothers were asked whether or not menstrual bleeding had occurred ( *status quo* method). Exclusion criteria included the use of medication for the management of metabolic, endocrine, or cardiovascular disease or body mass, or participation in a weight management program, including exercise or nutritional interventions, within 6 months prior to the study.

The study was approved by the institutional ethics committee of the University of Rio de Janeiro State (CAAE: 91950618.8.0000.5259). All parents or legal guardians signed informed consent forms providing authorization for the children to participate in the study.

### Procedures

Each participant visited the laboratory three times on three separate days. On the first day, anthropometric measurements were carried out by the same investigator and included body mass, height, waist, and hip circumferences. Body mass and height were assessed, respectively, by digital balance scales (Welmy, São Paulo, SP, Brazil) and a stadiometer graded in millimeters (American Medical do Brasil, São Paulo, SP, Brazil). BMI was subsequently calculated as body mass (kg) divided by squared height (m^2^). Waist circumference was taken midway between the lowest rib and the top of the iliac crest. Hip circumference was taken at the widest diameter of the buttocks. The waist-hip ratio was computed by dividing the waist circumference (cm) by the hip circumference (cm). Body composition ( *i.e.* , fat-free mass [FFM] and fat mass [FM], and percentage body fat) was assessed by dual-energy x-ray absorptiometry (Hologic QDR 4500, Hologic, Bedford, MA, USA).

On the second and third visits, REE was determined in accordance with the recommendations of AEL ( 10 ): abstention of physical exercise, soft drinks, and caffeine in the 24 h preceding the assessment, and overnight fasting prior to the assessment. The participants were instructed to wake slowly, minimize movement, and promptly come to the laboratory by car or bus early in the morning, expending as little energy as possible and without breakfast (about 12 h of fasting). In the laboratory, after confirming their compliance with these conditions, the participants laid on a bed in the supine position with their heads supported by a pillow and were covered with a bedsheet. They were instructed to remain silent and awake and to maintain a spontaneous breathing rhythm while in a calm and thermoneutral environment (22 to 24 °C) with minimum disturbance and light. Under this environment, an acclimation period of 10 min was undertaken with indirect calorimetry to decrease the participant’s anxiety during the assessment, after which the REE was measured for 30 min. The REE assessments were always performed at the same time of the day (between 7 a.m. and 11 a.m.), and the second trial was repeated after a 48-72 h interval to determine test-retest reliability. In girls, the REE assessments were synchronized to the menstrual cycle (measurements were carried out before ovulation).

Breath-by-breath pulmonary gas exchanges and min ventilation were recorded using a CPX Ultima CardiO2 indirect calorimeter (Medical Graphics Corp, St. Paul, MN, USA) and a Model 7400 oronasal mask (Hans Rudolph, Kansas, MO, USA) equipped with a Prevent metabolic flow sensor (Medical Graphics Corp, St. Paul, MN, USA). Flow calibrations were performed using a syringe graduated for a 3 L capacity (Hans Rudolph, Kansas, MO, USA) at the beginning of each test day, and gas calibrations were performed using a certified standard mixture of oxygen (17.01%) and carbon dioxide (5.00%), balanced with nitrogen (AGA, Rio de Janeiro, RJ, Brazil) according to the manufacturer’s instructions. MGC Diagnostic Breeze Suite 8.1.0.54 SP7 software (Medical Graphics Corp., St. Paul, MN, USA) was used to calculate the means of ventilation variables every min. The data obtained from the indirect calorimetry assessment included the VO_2_, VCO_2_, and REE. The REE was calculated by the Weir equation ( 15 ) and reported as kcal/day.

### Data analysis

Statistical analyses were performed using IBM SPSS Statistics 22 (SPSS Inc., Chicago, IL USA). Data were summarized using means and standard deviations (SD). Cohen’s d effect sizes for mean differences were calculated and defined as small (0.20), moderate (0.50), and large (0.80) ( 16 ). Statistical differences between groups with healthy weight (GHW) and obesity (GO) were investigated using unpaired Student’s *t* test. REE was statistically adjusted for FFM as described elsewhere ( 17 ) to control for variation attributable to differences in body composition.

The 30 min of VO_2_, VCO_2_ and REE data for each trial were split into 5-min stationary time averages ( *e.g.* , 1^st^-5^th^ min, 6^th^-10^th^ min, etc.), which seems to provide an accurate representation of the 24-h total energy expenditure than any other time interval in healthy populations ( 18 ). Changes in VO_2_ (L/min), VCO_2_ (L/min), and REE (kcal/d) were then analyzed with a marginal model using the Mixed procedure, with Trial and Time included as within-subject factors. *Post hoc* pairwise comparisons with Sidak-adjusted *p* values were used to identify at which time point there was no significant change in VO_2_, VCO_2_, and REE. The SSt condition was based on the absence of statistical significance between the 5-min stationary time averages. Two-tailed statistical significance for all null hypothesis tests was accepted as *p* ≤ 0.05. Test-retest reliability was evaluated by the mean difference across trials, and the intraclass correlation coefficient (ICC) was calculated as a one-way random-effects model ( 19 ).

## RESULTS

Anthropometric and body composition profiles of the GHW and GO, as well as REE data collected under SSt are summarized in Table 1 . The two groups were similar only for age, height, and REE adjusted for FFM. As expected, body mass, BMI, waist-hip ratio, and fat percentage were significantly higher in the GO than the GHW ( *p* ≤ 0.01).

**Table 1:** Characteristics of the participants at baseline

Variables	Group with healthy weight (n = 12; 2 girls)	Group with obesity (n = 18; 5 girls)	t test	p value
	Mean ± SD	Mean ± SD		
Age (years)	14.8 ± 1.5	14.4 ± 1.5	0.69	0.50
Tanner stage (range)	4-5	4-5	-	-
Height (cm)	168.6 ± 11.3	163.8 ± 8.2	1.4	0.181
Body mass (kg)	64.1 ± 13.5	91.8 ± 18.1	4.5	<0.001
Fat-free mass (kg)	46.5 ± 11.2	51.1 ± 7.4	1.4	0.186
Fat mass (kg)	17.6 ± 10.2	40.7 ± 13.1	5.2	<0.001
Percentage body fat (%)	25.3 ± 11.8	43.7 ± 6.0	5.6	<0.001
Body mass index (kg/m^2^)	22.5 ± 3.6	34.1 ± 5.2	6.7	<0.001
Waist circumference (cm)	80.1 ± 10.9	105.0 ± 13.7	5.2	<0.001
Hip circumference (cm)	93.3 ± 8.9	114.2 ± 13.9	4.6	<0.001
Waist-hip ratio	0.85 ± 0.1	0.92 ± 0.1	2.6	0.015
Resting energy expenditure adjusted for fat-free mass (kcal/day)	1,096 ± 144	1,156 ± 95	1.1	0.301

Abbreviation: SD, standard deviation

**Figure 1:**
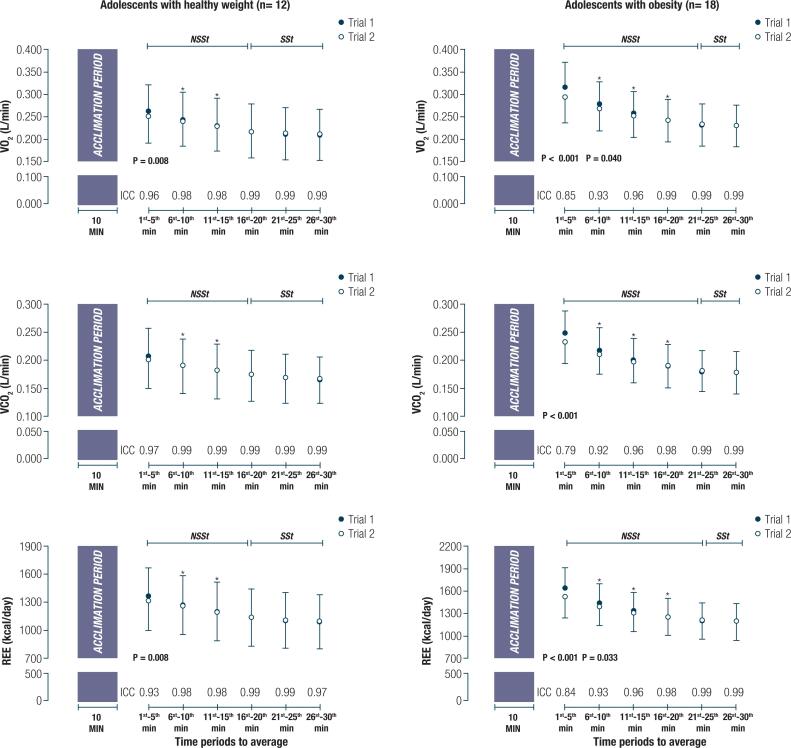
Mean ± standard deviation absolute resting oxygen uptake (VO_2_), carbon dioxide output (VCO_2_), and resting energy expenditure (REE) during two trials of 30-min data collection in adolescents with healthy weight and obesity. Abbreviations: NSSt, non-steady state; SSt, steady state. * Significantly lower than the previous value (p < 0.05). P values showing significant differences between Trial 1 vs. Trial 2. Intraclass correlation coefficient (ICC) for each time point between Trial 1 vs. Trial 2.

Table 2 shows the mean ± SD (95% confidence interval [CI]) values for VO_2_, VCO_2_, and REE for both trials of the 30-min assessment. Figure 1 shows the mean ± SD values for VO_2_, VCO_2_, and REE in graphical form, along with the test-retest reliability statistics for both trials of the 30-min assessment. Both groups had a significant change in VO_2_ (GHW: F = 89.2, *p* < 0.001; GO: F = 133.3, *p* < 0.001), VCO_2_ (GHW: F = 84.9, *p* < 0.001; GO: F = 175.8, *p* < 0.001), and REE (GHW: F = 103.6, *p* < 0.001; GO: F = 158.3, *p* < 0.001) during the 30-min assessment. *Post hoc* pairwise comparisons showed that for each of the three outcome variables, significant differences existed between consecutive means until the 20^th^ and 25^th^ min ( *i.e.* , non-steady-state period, NSSt) for the GHW and GO, respectively, after which no significant differences occurred ( *i.e.* , SSt condition). Regardless of the group ( *i.e.* , GHW *vs* . GO), when under SSt conditions, all assessments were characterized by a CV lower than 10% over a period of 5 consecutive min for either VO_2_ or VCO_2_ data. On the other hand, only 56% of the assessments achieved a CV < 10% under NSSt conditions in both groups.

**Table 2:** Mean ± standard deviation (95% confidence interval) absolute resting oxygen uptake (VO_2_), carbon dioxide output (VCO_2_), and resting energy expenditure (REE) during two trials of 30-min data collection in groups with healthy weight (GHW) and obesity (GO)

Variables	Trial	Time periods to average					
		1^st^-5^th^ min	6^th^-10^th^ min	11^th^-15^th^ min	16^th^-20^th^ min	21^st^-25^th^ min	26^th^-30^th^ min
**GHW**							
VO_2_ (L/min)	1	0.262 ± 0.054 (0.228-0.297)	0.243 ± 0.058 (0.206-0.280) *	0.231 ± 0.057 (0.194-0.267) *	0.219 ± 0.056 (0.183-0.254) *	0.212 ± 0.056 (0.176-0.247)	0.208 ± 0.056 (0.173-0.244)
	2	0.252 ± 0.057 (0.216-0.289)	0.241 ± 0.054 (0.207-0.275) *	0.228 ± 0.055 (0.194-0.263) *	0.217 ± 0.056 (0.182-0.253) *	0.213 ± 0.056 (0.177-0.249)	0.211 ± 0.056 (0.176-0.247)
VCO_2_ (L/min)	1	0.206 ± 0.048 (0.175-0.236)	0.190 ± 0.045 (0.161-0.219) *	0.181 ± 0.045 (0.153-0.210) *	0.174 ± 0.040 (0.149-0.200) *	0.167 ± 0.041 (0.142-0.193)	0.163 ± 0.040 (0.138-0.189)
	2	0.200 ± 0.050 (0.168-0.232)	0.190 ± 0.048 (0.159-0.220) *	0.180 ± 0.048 (0.150-0.210) *	0.173 ± 0.046 (0.144-0.202) *	0.168 ± 0.043 (0.141-0.195)	0.166 ± 0.042 (0.139-0.192)
REE (kcal/day)	1	1361 ± 287 (1179-1543)	1261 ± 301 (1070-1452) *	1199 ± 296 (1010-1387) *	1138 ± 285 (957-1320) *	1101 ± 286 (919-1282)	1081 ± 283 (901-1261)
	2	1314 ± 303 (1121-1506)	1251 ± 285 (1070-1433) *	1187 ± 289 (1003-1371) *	1132 ± 294 (946-1319) *	1106 ± 288 (923-1289)	1096 ± 285 (915-1277)
**GO**							
VO_2_ (L/min)	1	0.316 ± 0.055 (0.289-0.343)	0.278 ± 0.049 (0.254-0.302) *	0.258 ± 0.048 (0.234-0.281) *	0.242 ± 0.047 (0.219-0.266) *	0.231 ± 0.047 (0.207-0.254) *	0.229 ± 0.047 (0.206-0.252)
	2	0.293 ± 0.057 (0.264-0.321)	0.268 ± 0.049 (0.243-0.292) *	0.253 ± 0.049 (0.228-0.278) *	0.242 ± 0.048 (0.218-0.266) *	0.233 ± 0.048 (0.209-0.257) *	0.230 ± 0.047 (0.206-0.253)
VCO_2_ (L/min)	1	0.249 ± 0.039 (0.229-0.268)	0.218 ± 0.040 (0.198-0.238) *	0.201 ± 0.038 (0.182-0.219) *	0.189 ± 0.039 (0.170-0.208) *	0.179 ± 0.038 (0.160-0.198) *	0.178 ± 0.038 (0.159-0.197)
	2	0.233 ± 0.039 (0.213-0.252)	0.211 ± 0.036 (0.193-0.229) *	0.198 ± 0.038 (0.179-0.217) *	0.190 ± 0.039 (0.171-0.209) *	0.182 ± 0.038 (0.163-0.201) *	0.179 ± 0.039 (0.160-0.198)
REE (kcal/day)	1	1641 ± 272 (1450-1742)	1443 ± 254 (1276-1536) *	1335 ± 246 (1171-1420) *	1255 ± 246 (1087-1338) *	1195 ± 245 (1032-1276) *	1186 ± 246 (1024-1267)
	2	1525 ± 284 (1383-1663)	1391 ± 248 (1268-1515) *	1312 ± 254 (1187-1442) *	1256 ± 250 (1132-1381) *	1209 ± 248 (1085-1330) *	1191 ± 247 (1068-1311)

* Significantly lower than the previous value ( *p* < 0.05).

In the GHW, significant differences were observed between trials only at 5 min of assessment for VO_2_ (mean diff = 0.010 L/min, 95% CI = 0.003 to 0.017 L/min, *p* = 0.008, effect size [Cohen’s d] = 0.17) and REE (mean diff = 47 kcal/day, 95% CI = 12 to 82 kcal/day, *p* = 0.008, effect size [Cohen’s d] = 0.17), while in the GO, significant differences were observed until the 10-min time point for VO_2_ (1^st^ to 5^th^ min: mean diff = 0.023 L/min, 95% CI = 0.013 to 0.033 L/min, *p* < 0.001, effect size [Cohen’s d] = 0.42; 6^th^ to 10^th^ min: mean diff = 0.010 L/min, 95% CI = 0.000 to 0.020 L/min, *p* = 0.040, effect size [Cohen’s d] = 0.21) and REE (1^st^ to 5^th^ min: mean diff = 116 kcal/day, 95% CI = 69 to 163 kcal/day, *p* < 0.001, effect size [Cohen’s d] = 0.43; 5^th^ to 10^th^ min: mean diff = 51 kcal/day, 95% CI = 4 to 99 kcal/day, *p* = 0.033, effect size [Cohen’s d] = 0.21) or at 5 min for VCO_2_ (mean diff = 0.016 L/min, 95% CI = 0.009 to 0.023 L/min, *p* < 0.001, effect size [Cohen’s d] = 0.42), either as a main effect (VO_2_: F = 8.2, *p* = 0.005; VCO_2_: F = 5.9, *p* = 0.16; REE: F = 8.5, *p* = 0.004), or as an interaction with time (VO_2_: F = 3.7, *p* = 0.003; VCO_2_: F = 3.8, *p* = 0.002; REE: F = 4.2, *p* = 0.001). Although statistically significant differences between trials 1 and 2 were detected during the first 5 to 10 of assessment, the VO_2_ and VCO_2_ responses and the REE for each 5-min time point exhibited high test-retest reliability across trials in both groups ( *e.g.* , ICC ranging from 0.79 to 0.99).

## DISCUSSION

To the best of our knowledge, the present study is the first to identify the time needed to achieve SSt conditions for an accurate and reliable assessment of REE in adolescents with healthy weight and obesity. The major finding was that following an acclimation period of 10 min, at least 20 and 25 min of assessment were needed to achieve a SSt in the GHW and GO, respectively. Test-retest reliability was high and relatively constant from 20 min onwards in both groups, which means that a single-day assessment of REE would be sufficient in clinical and research settings.

Cunha and cols. ( 13 ) reported similar findings when studying REE assessment in 30 healthy men (age, 22 ± 3 years) who performed two 60-min trials in a supine position to determine the test-retest reliability. These authors showed, for example, that following an acclimation period of 10 min, significant differences existed between consecutive 5 min means until the 30 min time point, where the largest differences occurred between mean values taken at 1^st^-5^th^ min *vs* . 30^th^ min, after which no significant differences occurred for VO_2_, VCO_2_, and REE. In the present study, when considering averaged data across trials following an acclimation period of 10 min, there was a mean difference of 202 and 381 kcal/day between the estimated energy requirements calculated using the mean REE values taken at 1^st^-5^th^ min *vs* . 16^th^-20^th^ and 21^st^-25^th^ min under SSt for the GHW and GO, respectively. From a practical perspective, the adoption of a short-term protocol of 10 min, as supported by previous studies ( 11 , 12 , 20 ), would overestimate the REE by 15% and 32% in the GHW and GO, respectively. Taking into account that REE is defined as the lowest energy expenditure of a person at rest ( 18 ), it is not difficult to understand that such inherent error would have important practical consequences for clinical and research settings, especially among adolescents with obesity, who seem to require more time to achieve a SSt condition than those with healthy weight.

Mellecker and McManus ( 21 ) used a mask or mouthpiece/nose clip device to determine the REE of 23 healthy children (ages 7-12 years) during two 35-min trials and found no significant differences in REE when assessed after 10, 15, 20, or 25 min compared with 30 min for both devices, although the lowest variability in REE data had occurred after 20 min of assessment. In a cross-sectional study involving 76 Korean children and adolescents with healthy weight and 52 with obesity aged 7-18 years, Kim and cols. ( 22 ) evaluated the REE according to the main methodological criteria proposed by the systematic review of AEL ( 10 ). In contrast to the present study, the REE determination lasted only 15 min and the first 5 min were discarded. The mean ± SD REE values reported for the GHW and GO, respectively, were 1,231 ± 238 and 1,553 ± 307 kcal/day, which were similar to values observed by the present investigation at the 10^th^ min of assessment in both groups. Although Kim and cols. ( 22 ) stated that only the VO_2_ and VCO_2_ steady-state periods were selected, it is feasible to think that their abbreviated protocol may not be sufficient to permit a decrease in REE data. Interestingly, another study reported similar REE results in adolescents with healthy weight and obesity as those reported by Kim and cols. ( 22 ). For example, Rodríguez and cols. ( 23 ) found mean ± SD values of 1,391 ± 246 and 1,595 ± 277 kcal/day for the GHW and GO, respectively, after the participants underwent 30 min of assessment in a supine position and considering only VO_2_ and VCO_2_ data from SSt to determine the REE. Once again, the REE observed by Rodríguez and cols. ( 23 ) were close to the values reported by the present study at the 10^th^ min of assessment in GHW and GO. Despite the aforementioned studies supporting the idea that REE determination was calculated from VO_2_ and VCO_2_ data under SSt conditions, the authors did not state how long it took to attain a SSt ( 22 , 23 ).

Marra and cols. ( 24 ) evaluated the REE of 264 adolescents with obesity (109 boys aged 16.5 ± 1.3 years and 155 girls aged 16.2 ± 1.5 years) with BMI ranging from 30.0-70.0 kg/m^2^ and reported very high mean REE values in both boys (2,569 ± 459 kcal/day) and girls (2,018 ± 385 kcal/day) after applying a longer protocol characterized by 15 min of acclimation and a subsequent 45 min of assessment in a supine position (a total of 60 min). Notably, these REE values are greater than those reported in previous studies ( 22 , 23 ) and even twice the values found in the current study. Therefore, the following question remains: what could explain the elevated REE values reported by Marra and cols. ( 24 )? First, it is important to highlight that the authors did not describe which method was adopted to determine the REE ( *e.g.* , selection of a predefined time interval or SSt-based REE). The lack of information on how the REE was calculated may account in part for these higher values. Another issue is related to the prolonged protocol that may promote boredom and consequently fidgeting among adolescents. Tang and cols. ( 25 ) evaluated 20 children and adolescents with obesity aged 7-17 years who underwent a 4-week summer camp program. REE assessments were performed after a 12-h fast and a minimum 30-min rest period. Although these authors stated that assessments were recorded at 1-min intervals for a minimum of 15-30 min, it is unclear how the REE was calculated in terms of criteria and the time necessary for detection of a SSt, which is a similar issue found in the description of the methods for the study by Marra and cols. ( 24 ). In other words, high REE values were also reported pre- and post-summer camp ( *i.e* ., 1,936 ± 789 and 1,902 ± 575 kcal/day, respectively). Nevertheless, bearing in mind the different REE protocols and metabolic systems, as well as several factors that may underpin the interindividual variability of REE such as age, sex, body size, body composition, hormonal status, and a range of genetic and environmental influences, among others ( 5 , 26 ), the comparisons between results of the aforementioned studies should be viewed with caution.

Some limitations of the present study must be acknowledged. First, it was not possible to investigate the REE derived from different collection apparatus widely adopted in experimental or clinical applications, such as mouthpiece and ventilated canopy. Second, the sample size was relatively small ( *i.e.* , 12 adolescents with healthy weight and 18 with obesity). Although small sample sizes are common in this area of research, caution should always be exercised when interpreting the accuracy of parameter estimates derived from such sample sizes.

The present study, therefore, detected the existence of an optimal assessment duration of VO_2_ and VCO_2_ data for achieving a SSt condition, recommended to improve the accuracy of REE determination in adolescents with healthy weight and obesity. In conclusion, after completing a 10-min acclimation period, a minimum of a further 20 and 25 min of rest were necessary to obtain a VO_2_ and VCO_2_ SSt conducive to an accurate determination of REE in GHW and GO, respectively. On the basis of the main findings, the following recommendations are provided: a) initiate the REE assessment with 10 min of acclimation to decrease restlessness in adolescents; b) determine REE for a minimum of 20 min in GHW and 25 min in GO, until apparent VO_2_ and VCO_2_ SSts have been achieved; and c) determine REE for a further 5 min, with the average of this last 5 min of REE data being regarded as the REE.

## References

[1] Kansra AR, Lakkunarajah S, Jay MS (2021). Childhood and adolescent obesity: A review. Frontiers in Pediatrics.

[2] Afshin A, Forouzanfar MH, Reitsma MB, Sur P, Estep K, Lee A (2017). Health effects of overweight and obesity in 195 countries over 25 years. N Engl J Med.

[3] Kuczmarski RJ, Ogden CL, Guo SS, Grummer-Strawn LM, Flegal KM, Mei Z (2002). 2000 CDC Growth Charts for the United States: methods and development. Vital Health Stat 11.

[4] Lindberg L, Danielsson P, Persson M, Marcus C, Hagman E (2020). Association of childhood obesity with risk of early all-cause and cause-specific mortality: A Swedish prospective cohort study. PLoS Med.

[5] Hohenadel MG, Hollstein T, Thearle M, Reinhardt M, Piaggi P, Salbe AD (2019). A low resting metabolic rate in late childhood is associated with weight gain in adolescence. Metabolism.

[6] Ohlsson C, Bygdell M, Sondén A, Rosengren A, Kindblom JM (2016). Association between excessive BMI increase during puberty and risk of cardiovascular mortality in adult men: a population-based cohort study. Lancet Diabetes Endocrinol.

[7] Alberga AS, Prud’homme D, Sigal RJ, Goldfield GS, Hadjiyannakis S, Gougeon R (2017). Does exercise training affect resting metabolic rate in adolescents with obesity?. Appl Physiol Nutr Metab.

[8] Johnstone AM, Murison SD, Duncan JS, Rance KA, Speakman JR (2005). Factors influencing variation in basal metabolic rate include fat-free mass, fat mass, age, and circulating thyroxine but not sex, circulating leptin, or triiodothyronine. Am J Clin Nutr.

[9] Alcantara JMA, Sanchez-Delgado G, Martinez-Tellez B, Merchan-Ramirez E, Labayen I, Ruiz JR (2018). Congruent validity and inter-day reliability of two breath by breath metabolic carts to measure resting metabolic rate in young adults. Nutr Metab Cardiovasc Dis.

[10] Compher C, Frankenfield D, Keim N, Roth-Yousey L (2006). Best practice methods to apply to measurement of resting metabolic rate in adults: a systematic review. J Am Diet Assoc.

[11] Horner NK, Lampe JW, Patterson RE, Neuhouser ML, Beresford SA, Prentice RL (2001). Indirect calorimetry protocol development for measuring resting metabolic rate as a component of total energy expenditure in free-living postmenopausal women. J Nutr.

[12] Ventham JC, Reilly JJ (1999). Reproducibility of resting metabolic rate measurement in children. Br J Nutr.

[13] Cunha FA, Midgley AW, Monteiro W, Freire R, Lima T, Farinatti PT (2013). How long does it take to achieve steady state for an accurate assessment of resting VO2 in healthy men?. Eur J Appl Physiol.

[14] Cheng HL, Amatoury M, Steinbeck K (2016). Energy expenditure and intake during puberty in healthy nonobese adolescents: a systematic review. Am J Clin Nutr.

[15] Weir JB (1949). New methods for calculating metabolic rate with special reference to protein metabolism. J Physiol.

[16] Cohen J (1988). Statistical power analysis for the behavioral sciences.

[17] Ravussin E, Bogardus C (1989). Relationship of genetics, age, and physical fitness to daily energy expenditure and fuel utilization. Am J Clin Nutr.

[18] Irving CJ, Eggett DL, Fullmer S (2017). Comparing steady state to time interval and non-steady state measurements of resting metabolic rate. Nutr Clin Pract.

[19] Shrout PE, Fleiss JL (1979). Intraclass correlations: uses in assessing rater reliability. Psychol Bull.

[20] Borges JH, Guerra-Júnior G, Gonçalves EM (2019). Methods for data analysis of resting energy expenditure measured using indirect calorimetry. Nutrition.

[21] Mellecker RR, McManus AM (2009). Measurement of resting energy expenditure in healthy children. JPEN J Parenter Enteral Nutr.

[22] Kim MH, Kim JH, Kim EK (2012). Accuracy of predictive equations for resting energy expenditure (REE) in non-obese and obese Korean children and adolescents. Nutr Res Pract.

[23] Rodríguez G, Moreno LA, Sarría A, Pineda I, Fleta J, Pérez-González JM (2002). Determinants of resting energy expenditure in obese and non-obese children and adolescents. J Physiol Biochem.

[24] Marra M, Montagnese C, Sammarco R, Amato V, Della Valle E, Franzese A (2015). Accuracy of predictive equations for estimating resting energy expenditure in obese adolescents. J Pediatr.

[25] Tang Q, Ruan H, Tao Y, Zheng X, Shen X, Cai W (2014). Effects of a summer program for weight management in obese children and adolescents in Shanghai. Asia Pac J Clin Nutr.

[26] Herrmann SD, McMurray RG, Kim Y, Willis EA, Kang M, McCurdy T (2017). The influence of physical characteristics on the resting energy expenditure of youth: A meta-analysis. Am J Hum Biol.

